# Drying stability and permeability advantages of amorphous drug nanoparticles

**DOI:** 10.1186/s41120-026-00185-z

**Published:** 2026-07-24

**Authors:** Akshay Narula, Ajay Lale, Paroma Chakravarty, Na Li

**Affiliations:** 1 Department of Pharmaceutical Sciences, University of Connecticut, 69 North Eagleville Road Unit 3092, Storrs, CT 06269 USA; 2 Synthetic Molecule Pharmaceutical Sciences, Genentech, Inc., San Francisco, CA 94080 USA; 3 Institute of Materials Science, University of Connecticut, 97 North Eagleville Road Unit 3136, Storrs, CT 06269 USA; 4 Department Chemical & Biomolecular Engineering, University of Connecticut, 191 Auditorium Road, Unit 3222, Storrs, CT 06269 USA

**Keywords:** Amorphous nanoparticles, Stabilizer, Drying, Ionic interactions

## Abstract

The objective of this study was to develop stable amorphous drug nanoparticles with high drug loading and bioavailability advantages to overcome the drug-loading limitations of conventional amorphous solid dispersion (ASD) formulations. Amorphous drug nanoparticles were prepared *via* liquid-liquid phase separation in solution using various stabilizers, and their stability upon thermal drying and subsequent re-dispersion was evaluated. Zein, a stabilizer partitioning in the nanoparticle core alongside the active, was found to be highly effective in stabilizing said nanoparticles upon thermal drying. In contrast, most other stabilizers categorized as “wall materials”, required substantially higher amounts to achieve effective stabilization of the amorphous drug particles. Of these, the wall materials exhibiting ionic interactions with the drug, were found to markedly enhance nanoparticle stability during drying. Despite the use of stabilizers, nanoparticle drying stability varied with batch size, suggesting the need to consider the effects of both scale-up and process parameters for the development of this type of formulation. Subsequent membrane permeability studies with dried amorphous nanoparticles showed their membrane permeability to be comparable to that of fast-releasing ASDs. This phenomenon was found to be independent of drug-stabilizer ionic interactions in the nanoparticles. These results confirm amorphous drug nanoparticles to be an effective strategy for overcoming drug-loading constraints in ASD formulations without compromising bioavailability. Our results also provide insights into excipient-drug interactions and their impact on the drying stability of amorphous drug nanoparticles, which is critical for the development of stable, high drug-loading amorphous nanoparticle formulations.

**Graphical Abstract:**

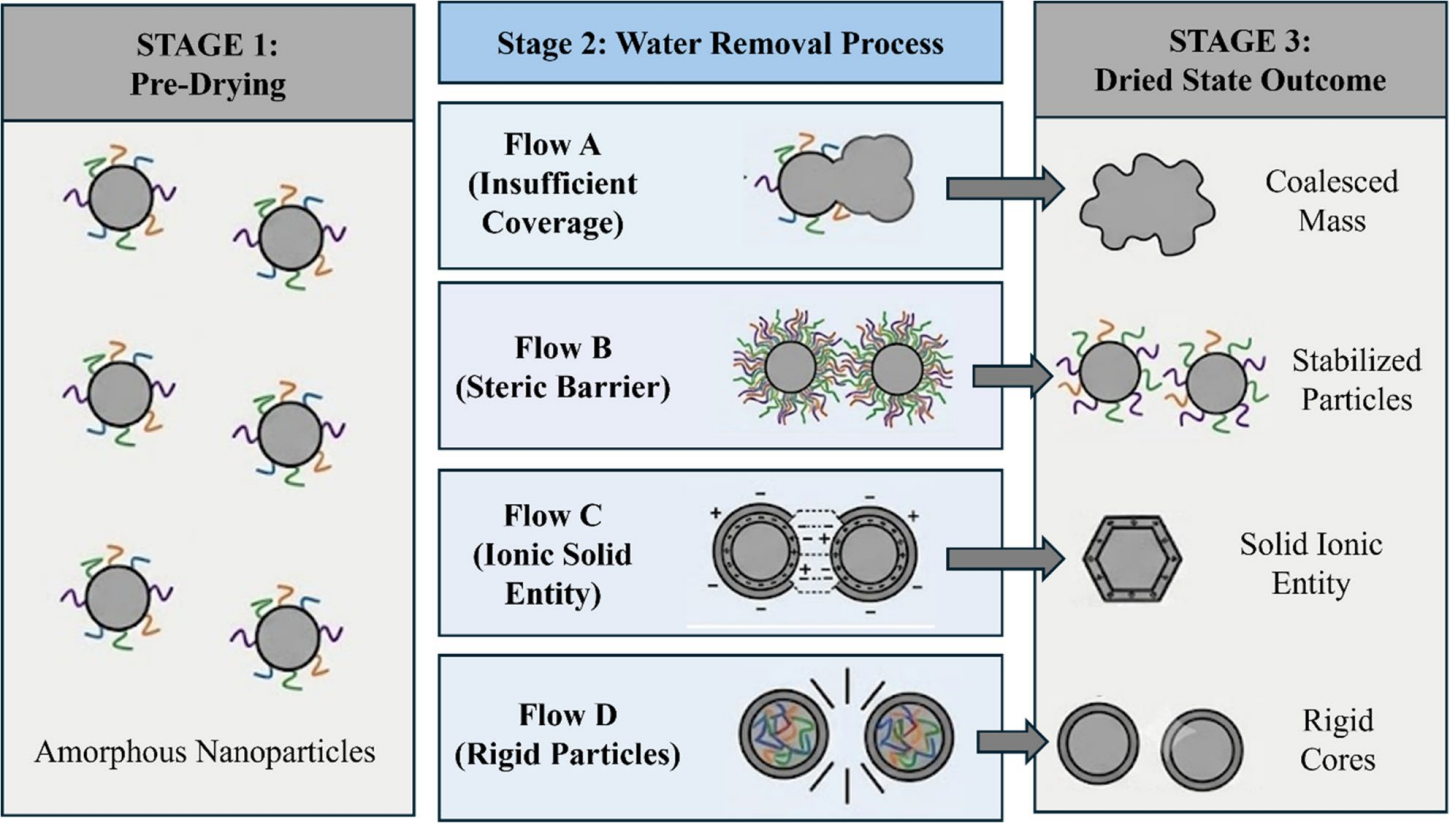

**Supplementary Information:**

The online version contains supplementary material available at 10.1186/s41120-026-00185-z.

## Introduction

A key challenge in the pharmaceutical industry is improving the aqueous solubility of poorly water-soluble drugs to enhance their absorption and therapeutic potential (Lipinski 2002). Amorphous solid dispersions (ASDs) comprising of a molecular dispersion of a drug and a suitable polymer, have been proven to be highly effective in addressing poor solubility issues (Baghel et al. 2016) as evidenced by the increasing numbers of FDA-approved drug products in recent years (Moseson et al. 2024). At low drug-loadings, ASDs undergo rapid and complete dissolution, driven by the fast dissolution of the hydrophilic polymer matrix (Saboo et al. 2020a, b). However, at high drug loadings, ASDs often exhibit slow and incomplete drug release, resulting in limited drug absorption (Saboo et al. 2020a, b; Deac et al. 2022). This bottleneck complicates the delivery of high therapeutic doses of poorly water-soluble drugs, often resulting in manufacturing challenges, oversized dosage forms, and reduced patient compliance.

To address the drug loading limitations of ASD formulations, several strategies have been explored. The addition of surfactants or plasticizers has been shown to improve drug release from ASDs (Mosquera-Giraldo et al. 2018; Correa-Soto et al. 2022), particularly for drugs with high glass transition temperatures (T_g_s). However, this approach represents a double-edged sword, as it carries the risk of compromising the physical stability of the amorphous drug (Wang et al. 2005; Janssens et al. 2008; Saboo et al. 2021; Correa-Soto et al. 2023). In addition, particle engineering strategies, such as the formation of hierarchy particles through surface coating with a hydrophilic polymer (Hiew et al. 2023), have proven to be highly effective in enhancing particle wetting and drug release. Nevertheless, this approach only applies to ASDs made with polymers that don’t dissolve in the coating medium. Forming high drug loading amorphous drug nanoparticles *via* liquid-liquid phase separation (LLPS) has emerged as a promising strategy to overcome the low drug loading issue of ASD formulations (Armstrong et al. 2023; Purohit et al. 2024). When the concentration of a dissolved solute exceeds its miscibility limit in water, liquid-liquid phase separation occurs. For poorly soluble drugs, this process often results in spontaneous formation of drug-rich amorphous nanoparticles, typically varying in size between ~ 200 nm and a few microns (Tachibana and Nakamura 1965; Brick et al. 2003; Ilevbare et al. 2013; Ilevbare and Taylor 2013; Indulkar et al. 2022). In addition to fast dissolution rates owning to their high surface area, these amorphous nanoparticles serve as drug reservoirs, maintaining the maximum achievable supersaturation (Indulkar et al. 2016) and potentially providing absorption advantages far beyond traditional solubility limits of the drug (Sugano 2010; Yang et al. 2024; Sharma et al. 2025). Enhanced absorption provided by amorphous drug nanoparticles is achieved by reducing the diffusional resistance of the aqueous boundary layer, thereby facilitating more efficient drug passage through absorption sites (Sugano 2010).

For the effective development of these amorphous drug nanoparticles into a feasible pharmaceutical formulation, it is essential to convert the dilute aqueous nanosuspension into a stable dry powder form suitable for delivery. Recent studies, including those by Matteucci et al. and Melzig et al., have highlighted advancements in producing high-potency amorphous nanoparticles through controlled precipitation, yet challenges remain in achieving successful re-dispersibility from the dry state (Matteucci et al. 2008; Melzig et al. 2018). Additionally, it often requires stabilizers in quantities much greater than the drug itself, which can limit the overall drug loading capacity in order to achieve redispersibility (Lee 2003; Melzig et al. 2018). For amorphous drug nanoparticles, their intrinsic softness (Sangtani et al. 2017) and amorphous state present multi-prong stability challenges in formulation development. Our previous study indicated coalescence as the dominant destabilization mechanism, while Ostwald ripening plays a negligible role during thermal drying of amorphous drug nanoparticles (Narula et al. 2024). In addition to particle size increase, the risk of drug crystallization in amorphous nanoparticles requires considerable attention (Jog et al. 2016) Therefore, it is critical to understand the impact of excipient selection on mitigating destabilization pathways (particle size increase and drug crystallization) during the drying process.

The goal of this research was to develop stable amorphous drug nanoparticles with good re-dispersibility and examine membrane permeability of these dried nanoparticles to assess their bioavailability advantage over conventional ASD formulations. For this purpose, model drugs with poor aqueous solubility, relatively low crystallization tendency, and varying T_g_s were selected. Specifically, clotrimazole (CTZ), felodipine (FDP), and tolnaftate (TNT) were chosen to screen for stabilizer performance owing to their differing ionization states at pH 6.5 and broad range of T_g_s. A wide variety of excipients, such as polymers, surfactants and emulsifiers, were evaluated as particle stabilizers. In addition, four weakly basic drugs, atazanavir (ATZ), bifonazole (BFZ), loratadine (LTD), miconazole (MCZ), and one neutral drug lopinavir (LPV), were used to evaluate the impact of drug-excipient ionic interactions on particle stability.

## Materials

### Selection of model compounds and excipients

**Fig. 1:**
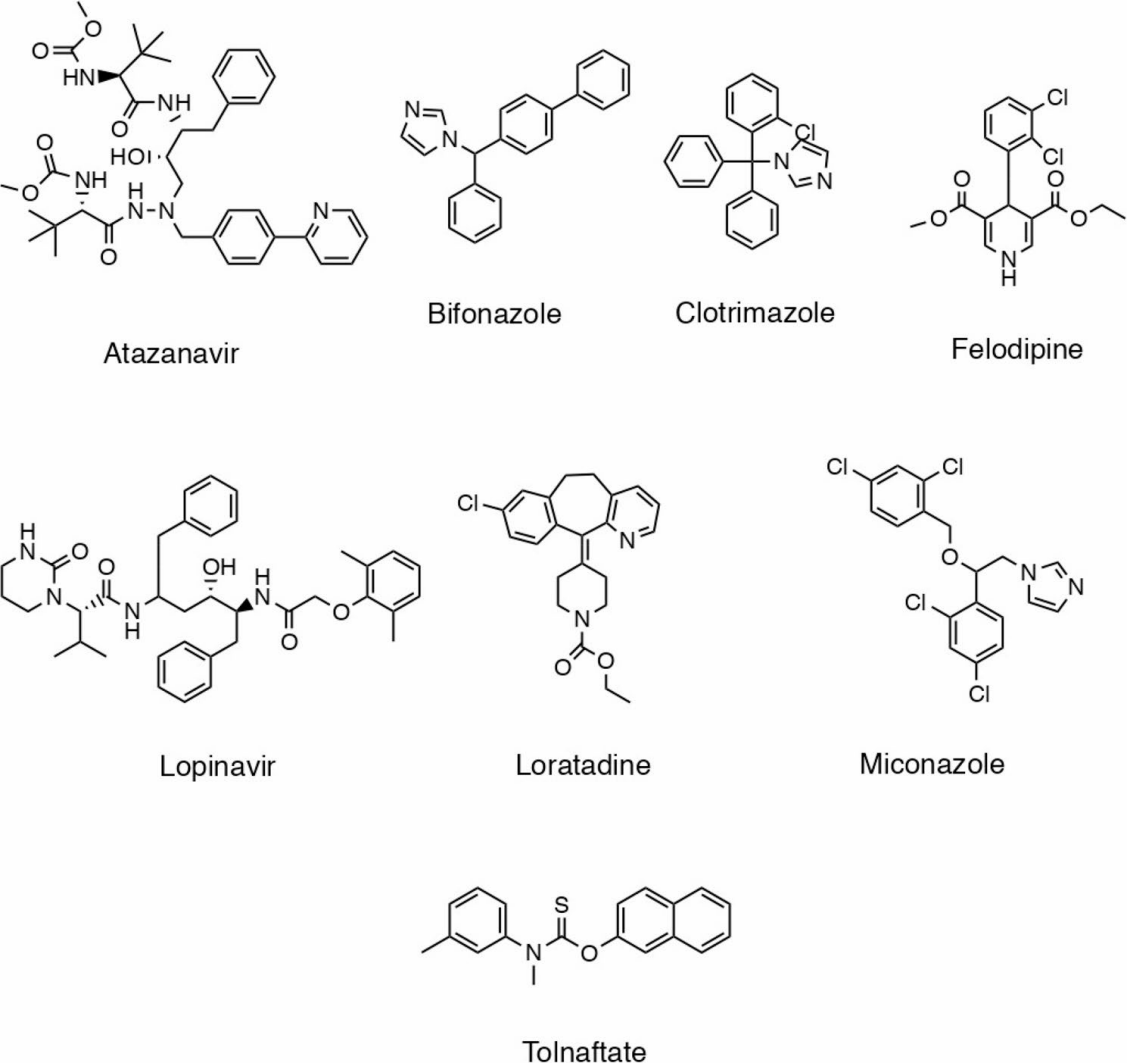
Molecular structure of model drugs

Fig. 1 displays the chemical structures of the selected model compounds. Their physicochemical properties are detailed in Table 1 along with the excipients selected. The study focuses on structurally diverse small molecule drugs such as ATZ, BFZ, CTZ, FDP, LPV, LTD, MCZ, and TNT known for their poor aqueous solubility and high logP values. At pH 6.5, BFZ, CTZ, MCZ, and LTD become ionized; whereas ATZ, LPV, FDP, and TNT remain unionized. This distinction allows for a systematic evaluation of excipient performance under varying ionization states. The variation in pK_a_, logP, T_g_ and crystallization tendencies enable the evaluation of how excipients stabilize nanoparticles under different conditions. ATZ and LPV were obtained from ChemShuttle (Wuxi, China). CTZ, BFZ, FDP, LTD, and TNT were sourced from Sigma-Aldrich (St. Louis, MO while MCZ was purchased from Fisher Scientific (Ward Hill, MA).

**Table 1:** Physicochemical properties of model drugs and stabilizers

Model compound	pK_a_	LogP	T_g_s (ºC, dry and wet)
Model drugs			
Atazanavir (ATZ)	4.52 (Indulkar et al. 2015), basic	5.2 (Di et al. 2012)	104 (Indulkar et al. 2015), 51 (Indulkar et al. 2015)
Bifonazole (BFZ)	5.72 (Popović and Čakar 2004), basic	4.21 (Wong et al. 2004)	17 (Baird et al. 2010), 8.3 ± 3.1*
Clotrimazole (CTZ)	5.89 (Indulkar et al. 2015), basic	6.1 (Bhide et al. 2020)	28 (Jacob et al. 2020), 19.3 (Narula et al. 2024)
Felodipine (FDP)	Neutral	3.86 (Yusuf et al. 2014)	44.7 ± 1.8 (Saboo et al. 2021),36.46 ± 2.88*
Lopinavir (LPV)	Remains undissociated at physiological pHs	4.7 (Yu et al. 2020)	69 (Li and Taylor 2018), 42.2 ± 1.8 (Narula et al. 2022)
Loratadine (LTD)	5.52 (Li et al. 2015), basic	5.2 (El-Sherbiny et al. 2007)	32 (Viciosa et al. 2020), 19.8 ± 1.6*
Miconazole (MCZ)	6.50 (Beggs 1992), basic	6.1 (Hussain et al. 2023)	1.65 (Six et al. 2001)
Tolnaftate (TNT)	Remains undissociated at physiological pHs	5.4 (Völgyi et al. 2008)	7.5, 4.5 ± 0.5*
Excipients			
Casein	4.9 (Swaisgood 2003), acidic	NA	144 (Kalichevsky et al. 1993), NA
Eudragit^®^ L100 (EUD)	4.45 (Barbosa et al. 2019), acidic	NA	195 (Parikh et al. 2016), NA
Gum Arabic	NA, NA	NA	125 (Vasile et al. 2020), NA
HPMC E5 (HPMC)	NA, neutral	NA	209 (Li and Taylor 2016), NA
Zein	NA, NA	NA	164 (Di Gioia et al. 1999), NA

*NA* not applicable

*RT* room temperature

*Values were experimentally measured in-house

For nanoparticle stabilization, the following excipients were chosen:

- Eudragit^®^ L100 (EUD): a methacrylic acid based anionic polymer with a high T_g_ of 195 °C (Parikh et al. 2016), was selected for its minimal partitioning into drug particles (Ueda and Taylor 2020). EUD forms a steric barrier to prevent coalescence and stabilizes drug nanoparticles through electrostatic interactions (Mason et al. 2006; Ueda and Taylor 2020).
- Hydroxypropyl methylcellulose (HPMC): this cellulose derivative with a high T_g_(Li and Taylor 2016) causes steric repulsion, which helps maintain the stability of colloidal dispersions (Ueda and Taylor 2020).
- Casein: This natural surfactant found in milk offers a unique mechanism of stabilization *via* the formation of solid particles that encase emulsion droplets (Pickering emulsion), thereby enhancing their mechanical barrier properties.
- Gum Arabic: This excipient acts as an effective emulsifying agent and also serves as a coating material, preserving the integrity and stability of emulsion droplets through rapid drying and solid barrier formation (Pérez-Alonso et al. 2003). Its unique glycoprotein structure enables the hydrophobic polypeptide chains to adsorb and anchor the molecules onto the particle surface, while the carbohydrate part inhibit flocculation and coalescence through electrostatic and steric repulsions (Garti and Reichman 1993; Buffo and Reineccius 2000).
- Zein: This high T_g_ excipient imparts mechanical strength for increased particle rigidity and reduces Oswald ripening rate (Tortorella et al. 2021).

Eudragit^®^ L100 (EUD) was a generous gift from Evonik Industries (Parsippany-Troy Hills, NJ), and Methocel E5 (HPMC) from Dow Chemical Company (Midland, MI). Gum Arabic was acquired from MP Biomedicals (Solon, Ohio), while casein was purchased from Fisher Scientific (Ward Hill, MA). Zein was obtained from Tokyo Chemical Industry (Cambridge, MA).

### Preparation of buffer solutions

A 50 mM sodium phosphate buffer at pH 6.5 was prepared to simulate the physiological pH of the human intestinal tract, by dissolving 3.186 g of sodium phosphate dibasic dihydrate and 4.434 g of sodium phosphate monobasic monohydrate in one liter of reverse osmosis (RO) water. Similarly, a 50 mM sodium phosphate buffer at pH 8.0 was prepared to provide a completely unionized condition for the drug by dissolving 7.66 g of sodium phosphate dibasic dihydrate and 0.963 g of sodium phosphate monobasic monohydrate in one liter of water. RO water with a resistivity value of 18 MΩ or higher was produced using a deionization system in the lab and used for all buffer preparations.

## Methods

### Preparation of heat-dried amorphous drug nanoparticles and reconstitution

To counteract rapid Ostwald ripening due to enhanced solute solubility, the antisolvent method was used to prepare amorphous drug nanoparticles with a solvent content below 2% (v/v). In this procedure, the drug was initially dissolved in a chosen solvent. The drug solution was then slowly titrated into the antisolvent, allowing controlled precipitation with the help of stabilizers. The buffer solution was stirred continuously at 1000 rpm and room temperature while the stock solution was added. Water-soluble stabilizers were pre-dissolved in a phosphate buffer at desired concentrations, whereas insoluble stabilizers, such as zein, were dissolved in the solvent stream alongside the drug.

Amorphous drug nanoparticle suspensions produced through the antisolvent technique were dried in a scintillation vial using a rotary evaporator (BUCHI Rotavapor^®^ R-300, Essen, Germany) at 45 °C and a pressure of 150 mbar. The resulting film obtained upon drying was reconstituted in phosphate buffer and vortexed for 1 min. The redispersibility of the nanoparticles was subsequently assessed via dynamic light scattering (DLS) analysis, and absence of crystallization was evaluated using PLM.

### Preparation of freeze-dried amorphous nanoparticles

Fast releasing amorphous solid dispersions (ASDs) containing 10% LPV (neutral drug) by weight with HPMC as the polymer were prepared to obtain solvent-free freeze-dried amorphous drug nanoparticles for permeability studies. A solution of LPV and HPMC in methanol and dichloromethane (1:1 v/v) was prepared and subjected to rapid solvent evaporation for ~ 5 min using a rotary evaporator (BUCHI Rotavapor^®^ R-300, Essen, Germany) at 55 °C and 10 mbar to obtain amorphous solids. Residual solvents were removed by placing the samples in a vacuum oven at 25 °C overnight. Polarized light microscopy (PLM) was used to confirm the absence of crystallinity. The resulting ASD was then dissolved in buffer and vortexed thoroughly to ensure uniform wetting and complete dissolution. Subsequently, the sample was flash-frozen by adding liquid nitrogen to rapidly stabilize the amorphous nanoparticles.

The frozen samples were lyophilized using a benchtop lyophilizer (Labconco, Catalog no. 700201000) to remove water. It should be noted that lyophilization was considered here as an experimental-scale preparative method to yield stable, solvent-free particles for in vitro permeability evaluation, rather than to formulate a final dosage form. All samples were freshly prepared prior to membrane permeability experiments.

### Wet glass transition temperature (wet T_g_) determination

The wet T_g_ represents the T_g_ of water-saturated amorphous drug nanoparticles formed in situ. Differential scanning calorimetry (DSC) was employed to measure the wet T_g_ for the model drugs studied.

For the neat drug, crystalline powder was heated to its melting point and subsequently quenched on ice to produce the amorphous form. Approximately 5–10 mg of the amorphous drug was weighed into open hermetic DSC pans and equilibrated at 97% relative humidity using a saturated potassium sulphate solution for 48 h. After equilibration, the samples were sealed in hermetic pans and analyzed using a Q-20 series DSC instrument (TA Instruments, New Castle, DE). A heating ramp of 0–90 °C at 10 °C/min was applied during the DSC runs. DSC thermograms were analyzed with TA Universal Analysis software (TA Instruments, New Castle, DE). All experiments were performed in triplicate to ensure reproducibility.

### Particle size measurement

The particle size of amorphous drug nanoparticles was evaluated using dynamic light scattering (DLS). For in situ generated nanoparticles, the suspensions prepared in 50 mM sodium phosphate buffer (pH 6.5) were immediately transferred to disposable polystyrene cuvettes without further dilution. For dried nanoparticles, a predetermined volume of the same buffer was added to the vial to achieve the target concentration. The sample was then vortexed for 1 min for complete detachment of the film from the inner vial walls and to disperse any transient agglomerates before being immediately transferred to the cuvettes. Measurements were conducted at 25 °C using a Malvern nanoZS Zetasizer (Malvern Instruments, Westborough, MA). Each measurement was conducted in triplicates.

### Microstructure characterization by TEM-EDX

Transmission Electron Microscopy (TEM) imaging, Scanning TEM (STEM) - High-Angle Annular Dark-Field (HAADF), and Energy-Dispersive X-Ray Spectroscopy (EDX) analysis were performed using an FEI Talos F200X electron microscope, operating at 200 kV, and a SuperX silicon drift detector for elemental analysis. Ultrathin carbon-coated copper TEM grids (300 mesh, 3–4 nm carbon thickness) were used for sample preparation. A small aliquot of the nanoparticle suspension was pipetted onto the grid, spread evenly by slightly tilting the grid, and allowed to air dry before imaging.

Dark-field TEM micrographs were acquired to capture nanoparticle morphology and structural changes. EDX analysis was conducted to determine elemental composition. For comparing two distinct stabilization processes, three representative systems were selected for microstructure evaluation: CTZ-EUD and BFZ-EUD were selected to represent stabilization through ionic interactions (weakly basic drugs with an anionic polymer), while LPV-HPMC was selected to represent steric stabilization. EUD was used as the stabilizer for CTZ and BFZ to stabilize the particles throughout thermal drying. For LPV, the drug crystallized upon drying at a larger scale, and therefore HPMC was used as an alternative drying stabilizer. All TEM samples were prepared *via* rotary evaporation.

For the EDX analysis of CTZ-EUD samples, chlorine was used as a marker to probe the drug CTZ. In case of BFZ-EUD and LPV-HPMC samples, nitrogen served as the marker for BFZ and LPV. Sodium was used to map the distribution of the buffer solution. The beam spot size was carefully optimized to balance spatial resolution with X-ray count rate. Fast Fourier Transform (FFT) analysis was performed using Gatan Microscopy Suite Software to evaluate the crystallinity or amorphous nature of the samples. To ensure reproducibility and reliability, three separate TEM grids were prepared and analyzed for each sample.

### Flux measurements

Franz diffusion cells (PermeGear, Hellertown, PA) were used with a 5 mL apical (donor) and a 5 mL basolateral (receiver) compartment, separated by a 15 mm diameter orifice. A mesh screen divided the compartments, and each side was stirred independently, as described by Stewart et al (Stewart et al. 2017). To the donor chamber, a 50 mM sodium phosphate buffer (pH 6.5 or pH 8.0) containing EUD as a stabilizer was added for the formation of in situ amorphous particles. (see schematic representation of the permeation setup in Fig. S2, Supporting Information). The amorphous nature of the particles in the donor chamber was routinely confirmed by the absence of birefringence using PLM. For dried particles, the reconstituted nanoparticle suspensions were introduced directly into the donor chamber. For establishing a direct correlation between the confirmed amorphous microstructures and their resulting bioperformance, the exact same three representative model systems considered in the TEM evaluation (CTZ-EUD, BFZ-EUD, and LPV-HPMC) were selected for flux measurements.

The receiver chamber was filled with the same buffer solution, with 3% (w/v) BSA. A hydrophilic PVDF membrane (0.45 μm pore size, 25 mm diameter) from Millipore Sigma (Burlington, MA) was used as a filter support, and 150 µL of 15% (w/v) soy lecithin dissolved in dodecane was applied to form a lipophilic barrier over the membrane pores. Prior to the permeation experiments, Franz cells and buffer solutions were pre-equilibrated at 25 °C. Diffusion started with the addition of a small amount of drug dissolved in methanol or DMSO to the donor compartment, while ensuring that the volume of organic solvents in the buffer solution was kept at or below 2% (v/v) to minimize Ostwald ripening due to increased solubility (Liu et al. 2007) unless otherwise noted. For generating amorphous drug particles, a stirring rate of 1000 rpm was maintained unless otherwise specified. Both compartments were stirred at 150 rpm. Samples of 200 µL were withdrawn from the donor chamber at the start (0 h) and after 3 h to measure initial and final drug concentrations. Additionally, 200 µL samples were taken from the receiver chamber at 1, 2, and 3 h, with fresh buffer added to maintain the receiver volume.

For extracting the total drug content and precipitate BSA and lecithin prior to HPLC analysis, each 200 µL sample withdrawn from the chambers was mixed with 600 µL of acetonitrile. This high ratio of organic solvent was required for the immediate and complete dissolution of any intact nanoparticles into a molecular solution, eliminating the risk of crystallization during sample prepration. The mixture was vortexed for 10 s, and centrifuged at 16,500 rpm for 10 min using an Eppendorf 5430R centrifuge (Eppendorf, Hamburg, Germany). The supernatant was then analyzed by High Performance Liquid Chromatography (HPLC).

The total drug content in the receiver solution was calculated, accounting for the drug concentration in the sampling volume (200 µL). Drug content in the receiver was plotted over time, and the rate of drug permeation was calculated from the slope of this plot, representing mass flow. The flux was obtained by dividing the mass flow by the orifice area (15 mm diameter). Each experiment was repeated at least three times.

### High Performance Liquid Chromatography (HPLC)

An Agilent 1260 Infinity series HPLC system (Agilent Technologies, Santa Clara, CA), equipped with an Agilent Eclipse C18 column (150 mm length, 4.6 μm particle size), was utilized. The analyses were conducted using an isocratic flow at 1 mL/min, with a total run time of 8 min for LPV and 11 min for BFZ, respectively. Detection was performed at a wavelength of 210 nm, with varied injection volumes for samples from donor and receiver sides. For LPV, the mobile phase included 60% acetonitrile and 40% water, with calibration ranges of 5–120 µg/mL (5 µL injection) and 0.025–5 µg/mL (100 µL injection). BFZ was analyzed using a mobile phase with 45% acetonitrile and 55% water, also containing 0.1% trifluoroacetic acid (v/v), with calibration curves set for ranges of 0–400 µg/mL (5 µL injection) and 0–5 µg/mL (80 µL injection). The retention times were 5.3 min for LPV and 5.7 min for BFZ.

## Results

### Particle stability

#### Impact of stabilizers

**Fig. 2:**
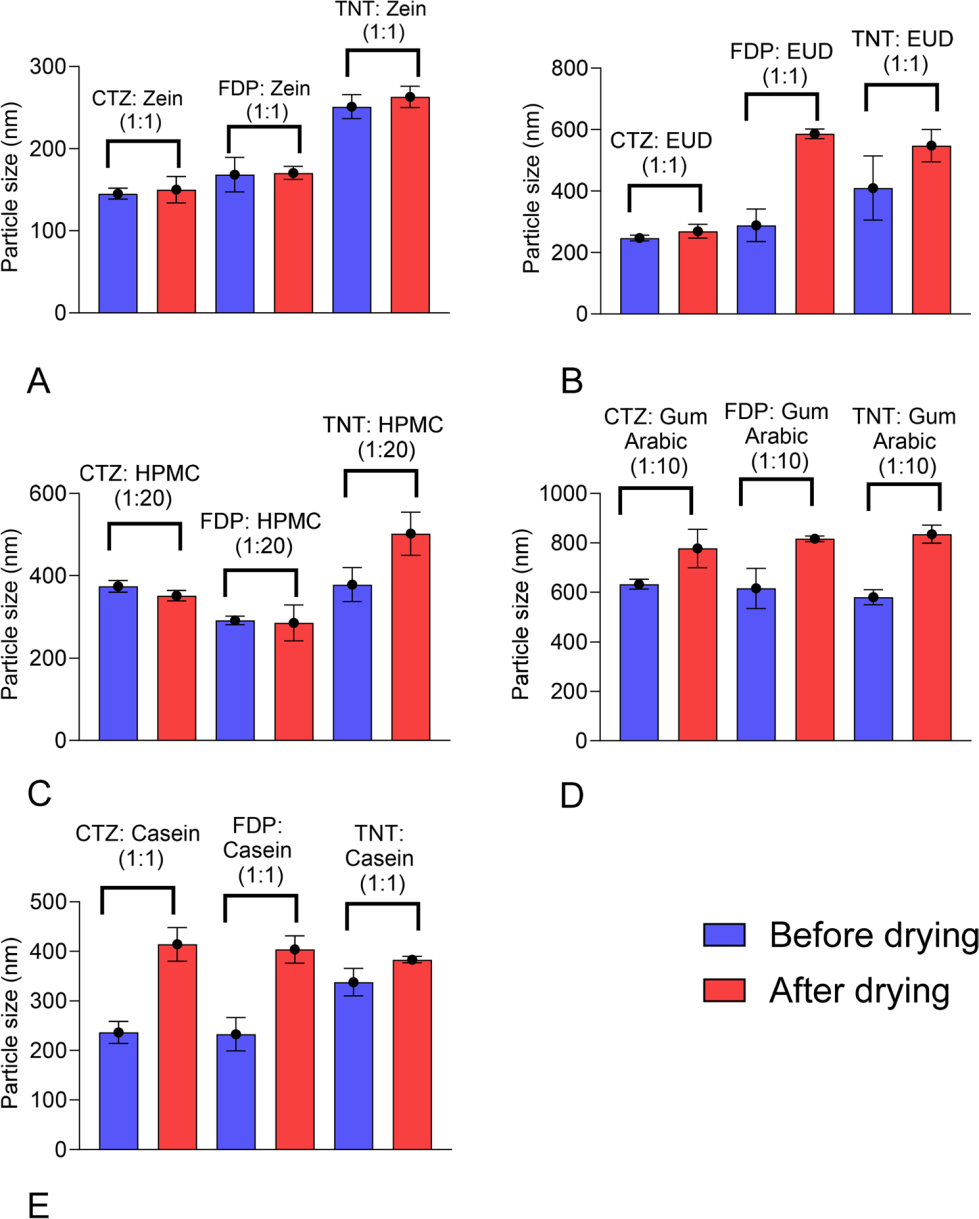
Impact of stabilizers on particle stability after drying for clotrimazole, felodipine and tolnaftate with **A**) zein, **B**) EUD, **C**) HPMC, **D**) gum Arabic, and **D**) casein as stabilizers (DLS data showed clean, monomodal distributions with good data quality. A more comprehensive data set with data of varying quality are provided in Table S1 in the Supporting Information)

As mentioned in the Introduction section, CTZ, FDP, and TNT were chosen for stabilizer screening experiments owing to their different ionization states at pH 6.5 and wide range of T_g_s (see Table 1), allowing evaluation of excipient performance across varied drug properties. As shown in Fig. 2A, amorphous drug particles were successfully stabilized throughout heat drying for all three drugs tested at a zein: drug ratio of 1:1. For EUD, FDP and TNT did not achieve stabilization at a 1:1 drug to EUD ratio (Fig. 2B). A series of increasing ratios of EUD detailed in Table S1 (Supporting Information) showed a similar trend. However, CTZ displayed marked stability at 1:1 drug to EUD ratio. Various drug: HPMC ratios, ranging from 1:1 to 1:20 as shown in Table S2 (Supporting Information) were evaluated, and a drug: HPMC ratio of 1:20 was needed to inhibit particle growth (Fig. 2C). Nevertheless, TNT particles still showed significant growth upon drying. For gum Arabic-stabilized systems, an optimized drug: gum Arabic ratio of 1:10 was selected through screening experiments detailed in Table S3 (Supporting Information). However, the particles still showed significant growth post-drying for all three drugs (Fig. 2D). For casein, a drug: casein ratio of 1:1 was selected based on preliminary results showing stabilization independent of casein concentration (Table S4, Supporting Information). The particle size of all three drugs stabilized by casein remained ~ 400 nm after drying (Fig. 2E). A conceptual schematic illustrating these distinct post-drying particle behaviors and stabilization mechanisms is provided in Fig. S3 (Supporting Information).

#### Impact of ionic interactions on particle stability

**Fig. 3:**
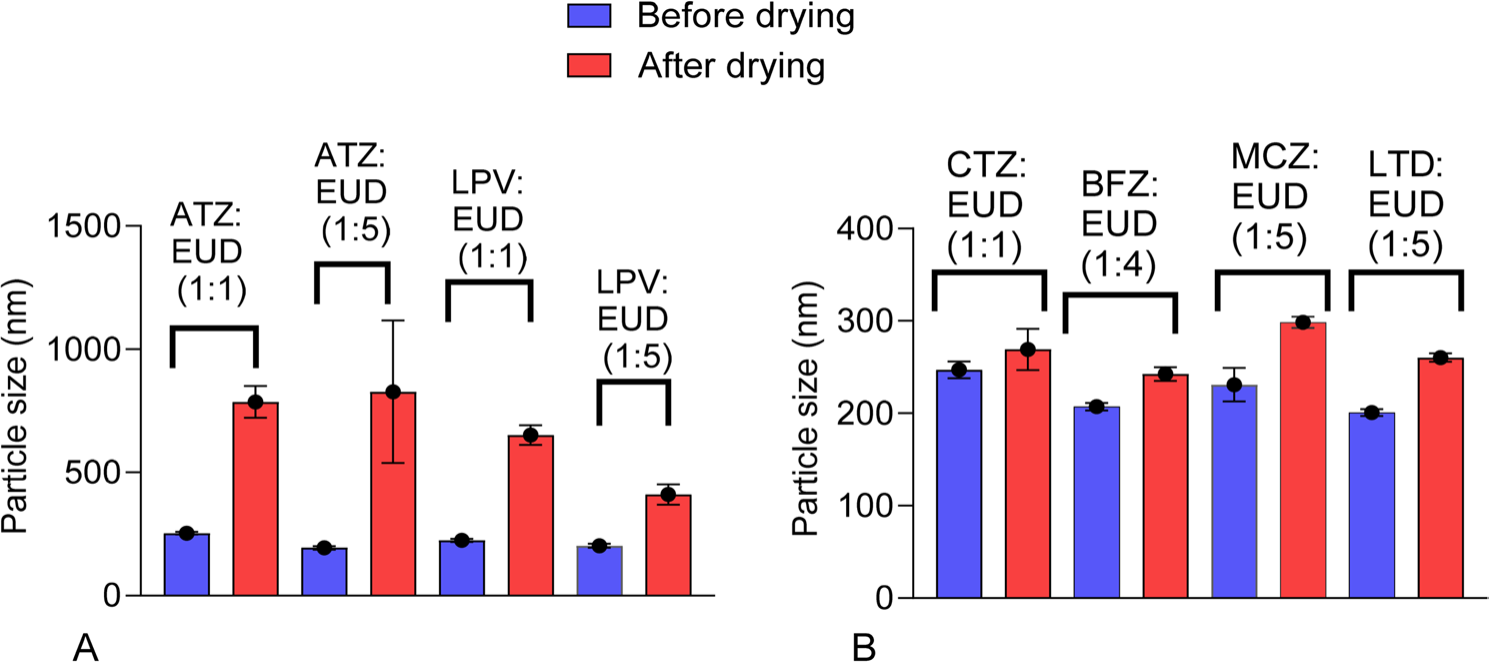
The size of **A**) atazanavir, lopinavir and **B**) clotrimazole, bifonazole, miconazole and loratadine amorphous nanoparticles before and after drying with EUD as a stabilizer (DLS data showed clean, monomodal distributions with good data quality for successfully stabilized formulations with PDI ranging from 0.05 to 0.35; formulations that failed to stabilize, ATZ: EUD and LPV: EUD, exhibited higher PDIs)

The size of amorphous nanoparticles stabilized with EUD was evaluated using both non-ionizable and ionizable drugs at pH 6.5, as shown in Fig. 3. ATZ (weakly basic) and LPV (neutral) exhibited significant particle growth during drying, regardless of the drug : EUD ratio (1:1 or 1:5). In contrast, several weakly basic drugs, including CTZ, BFZ, MCZ, and LTD, demonstrated enhanced particle stability, with minimal size growth observed after drying. Salt formation between the ionized drug and the anionic polymer likely contributed to the stabilization of particles during drying (see the mechanistic schematic in Fig. S1, Supporting Information). At the experimental pH of 6.5, the weakly basic drugs are partially ionized (protonated), while the carboxylate groups of the anionic polymer (e.g., Eudragit L100) are deprotonated. The resulting intermolecular ionic interactions (salt bridges) facilitate the formation of a dense, rigid polymer shell that prevents core coalescence, consistent with the findings of Narula et al. in solution (Narula et al. 2026).

#### Microstructure

**Fig. 4:**
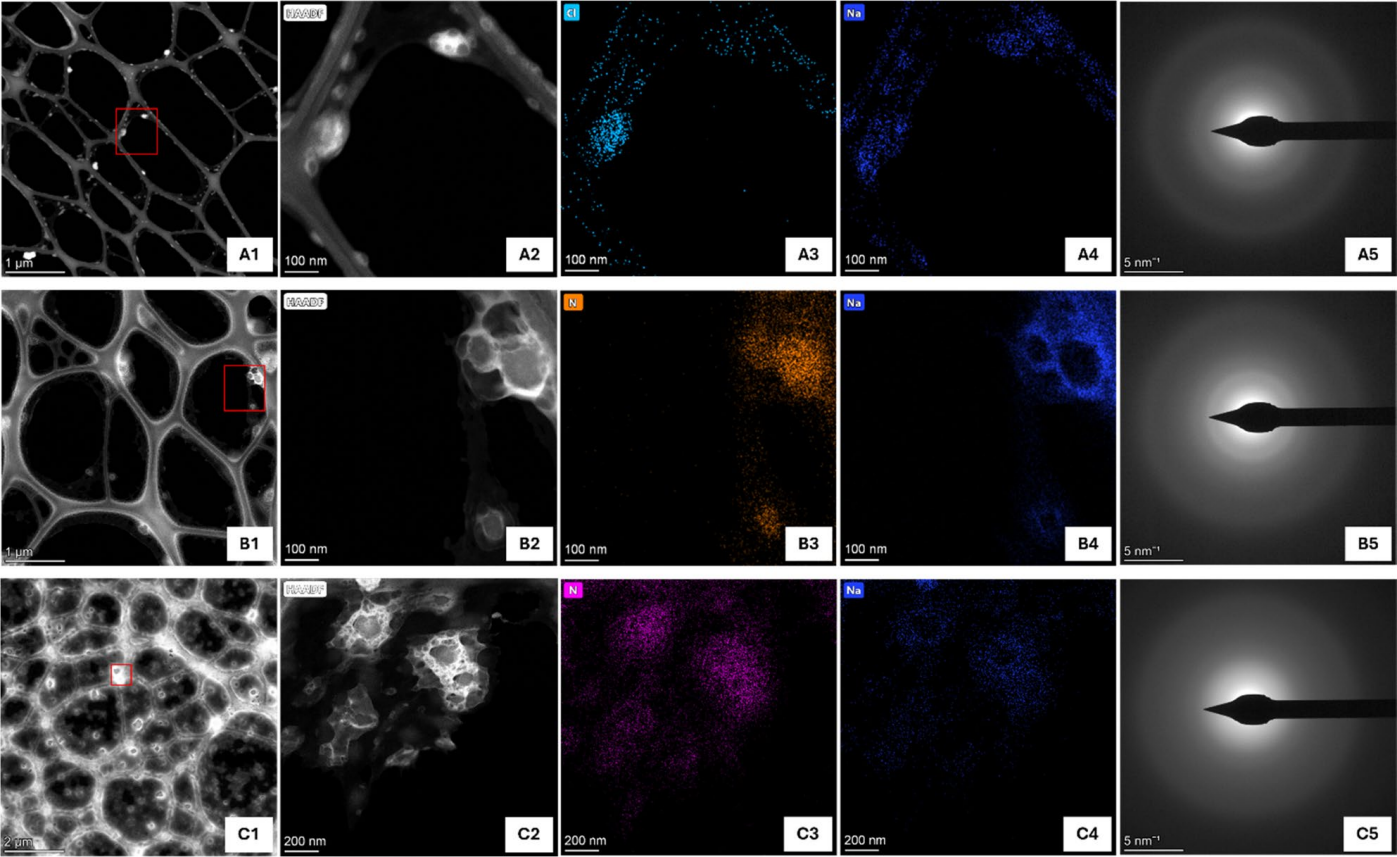
Microstructure analysis showing (1) TEM images, (2) STEM-HAADF images of nanoparticles, 3 and 4) EDX, and 5) diffraction analyses of **A**) clotrimazole, **B**) bifonazole, and **C**) lopinavir (the network structures are the TEM grids)

Microstructural characterization of dried CTZ-EUD, BFZ-EUD, and LPV-HPMC nanoparticles were performed through TEM, HAADF-STEM, EDX, and diffraction analyses. These analyses collectively showed the formation of nonaggregate, drug-rich, uniform amorphous nanoparticles in all three formulations (Fig. 4).

Low-magnification dark-field TEM images (Fig. 4A1, B1, C1) showed well-dispersed nanoparticulate structures dispersed within the TEM grids, with no evidence of extensive aggregation. The corresponding HAADF-STEM images (Fig. 4A2, B2, C2) represent higher-magnification views of the boxed regions in the dark-field images and were acquired after repeated STEM-EDX scans over 30 min. These regions were further scanned to determine their elemental distribution.

For CTZ-EUD (Fig. 4A), EDX mapping showed a distinct chlorine signal colocalized with the nanoparticle region, confirming the presence of CTZ-rich domains (Fig. 4A3). A sodium signal was also detected in the surrounding area and is attributed to dried phosphate buffer salts (Fig. 4A4). The corresponding diffraction pattern displayed a diffuse halo without discrete spots or rings (Fig. 4A5), indicating the absence of long-range crystalline order and confirming that the particles remained amorphous.

In case of BFZ-EUD (Fig. 4B), discrete nanoparticle domains were similarly observed. EDX analysis revealed a localized nitrogen signal associated with the particle-rich region, consistent with BFZ-rich drug particles (Fig. 4B3), whereas the sodium signal was assigned to residual buffer surrounding the particles (Fig. 4B4). The diffuse diffraction pattern again lacked sharp reflections (Fig. 4B5), supporting the amorphous nature of the BFZ-rich nanoparticles.

Moreover, LPV-HPMC formulation (Fig. 4C) showed numerous well-defined nanoparticle structures without obvious aggregation. Similarly, EDX mapping showed a clear nitrogen signal associated with these domains (Fig. 4C3), consistent with LPV-rich particles, while the sodium signal was attributed to residual phosphate buffer (Fig. 4C4). The corresponding diffraction pattern showed diffuse scattering (Fig. 4C5), confirming the absence of crystallinity.

### Membrane permeability

Drug nanoparticles possess the unique advantage of enhancing membrane permeability by moving in the aqueous boundary layer and releasing the drug at the surface of the membrane (Sugano 2010; Narula et al. 2022; Sinko et al. 2024; Yang et al. 2024; Sharma et al. 2025). However, it remains unknown whether the hydration state of these particles would alter particle re-dissolution rate and subsequent membrane permeability. Previously, ionic interactions between the drug and HPMCAS were found to negatively impact drug release from amorphous solid dispersions (ASDs), due to the formation of hydrophobic ion pairs and reduced hydration of the polymer (Bapat et al. 2024). Therefore, in this study, two weakly basic drugs, CTZ and BFZ (stabilized using EUD), as well as a neutral drug LPV (stabilized using HPMC due to crystallization issues found with EUD) were used to evaluate the impact of drying on their permeability advantage. Wet amorphous nanoparticles formed in situ, prepared using either methanol or DMSO stock solutions, were used as a control system.

For CTZ, at particle concentrations lower than 150 µg/mL, comparable flux values were obtained between dried and in situ particles. However, particle aggregation and crystallization were observed at higher particle concentrations for this system using DLS and PLM analysis, possibly due to the low polymer concentration (1:1 drug: polymer) used. Despite successful preparation of re-dispersible amorphous nanoparticles during excipient screening and small volumes of samples for TEM imaging (2 mL), scale-up for permeability evaluation studies (6 mL) required prolonged drying time (> 30 min) to remove larger volumes of continuous phase, which likely triggered drug crystallization.

**Fig. 5:**
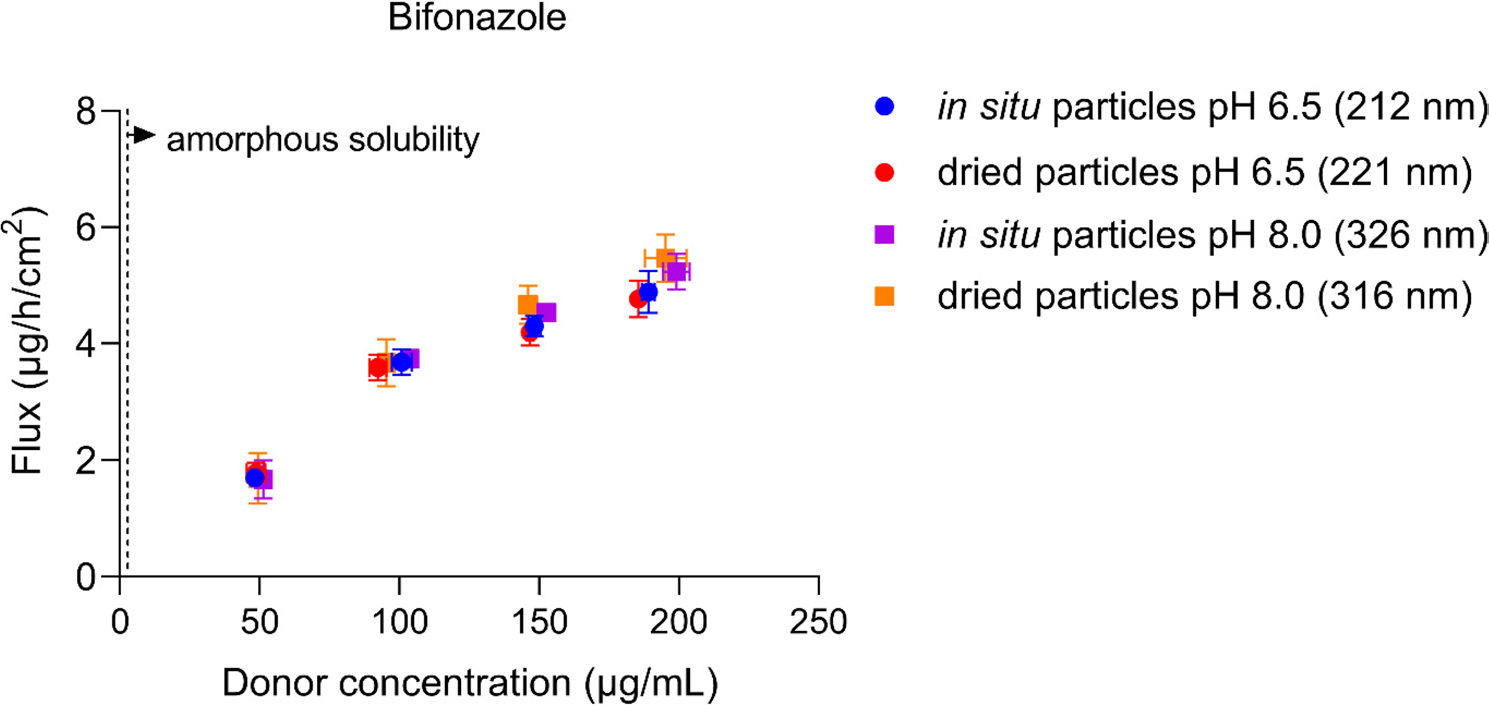
Membrane flux of BFZ from in situ and dried amorphous nanoparticles at pH 6.5 and pH 8.0

For the BFZ-EUD system, a 1:4 drug to polymer ratio was used to stabilize the particles. As shown in Fig. 5, both in situ and dried BFZ-EUD amorphous nanoparticles showed comparable flux at multiple concentrations tested. Additionally, we also evaluated pH 8.0 buffer as the dissolution media, where 99.5% of BFZ was unionized and ionic interactions between the drug and the polymer are negligible, to test its impact on particle re-dissolution. Increasing donor solution pH to 8.0 increased the measured hydrodynamic diameter of amorphous nanoparticles (~ 316–326 nm), yet flux remained similar to that obtained at pH 6.5, where 14.2% of BFZ was ionized. Also, at pH 8, the in situ and dried particles showed similar flux. Overall, these results indicated that drying and reconstitution did not measurably impact the flux of BFZ, and the net effect of drug ionization on particle re-dissolution and flux enhancement was insignificant for EUD stabilized systems.

**Fig. 6:**
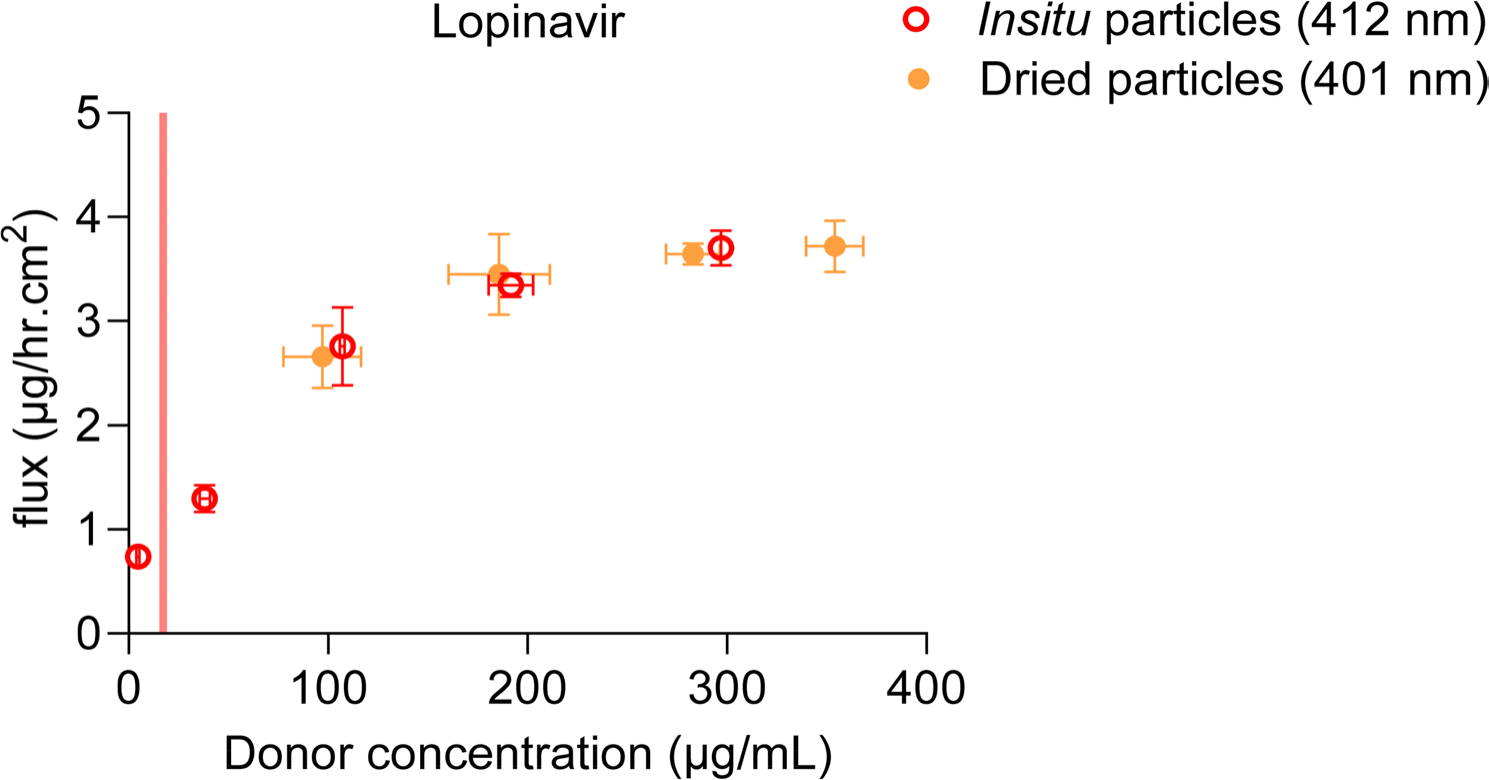
Membrane flux of LPV from in situ and dried amorphous nanoparticles

For the neutral drug LPV, scale-up challenges similar to those of CTZ, were observed during thermal drying, despite the use of a higher stabilizer ratio. Consequently, lyophilization was employed as an alternative drying method to produce amorphous drug nanoparticles. Fig. 6 shows the flux of LPV from in situ and dried amorphous particles. Both in situ particles (412 nm) and dried particles (401 nm) exhibited comparable flux values at all donor concentrations, indicating no significant impact of drying on the permeability advantages provided by these drug nanoparticles.

## Discussion

### Stabilizer selection

**Fig. 7:**
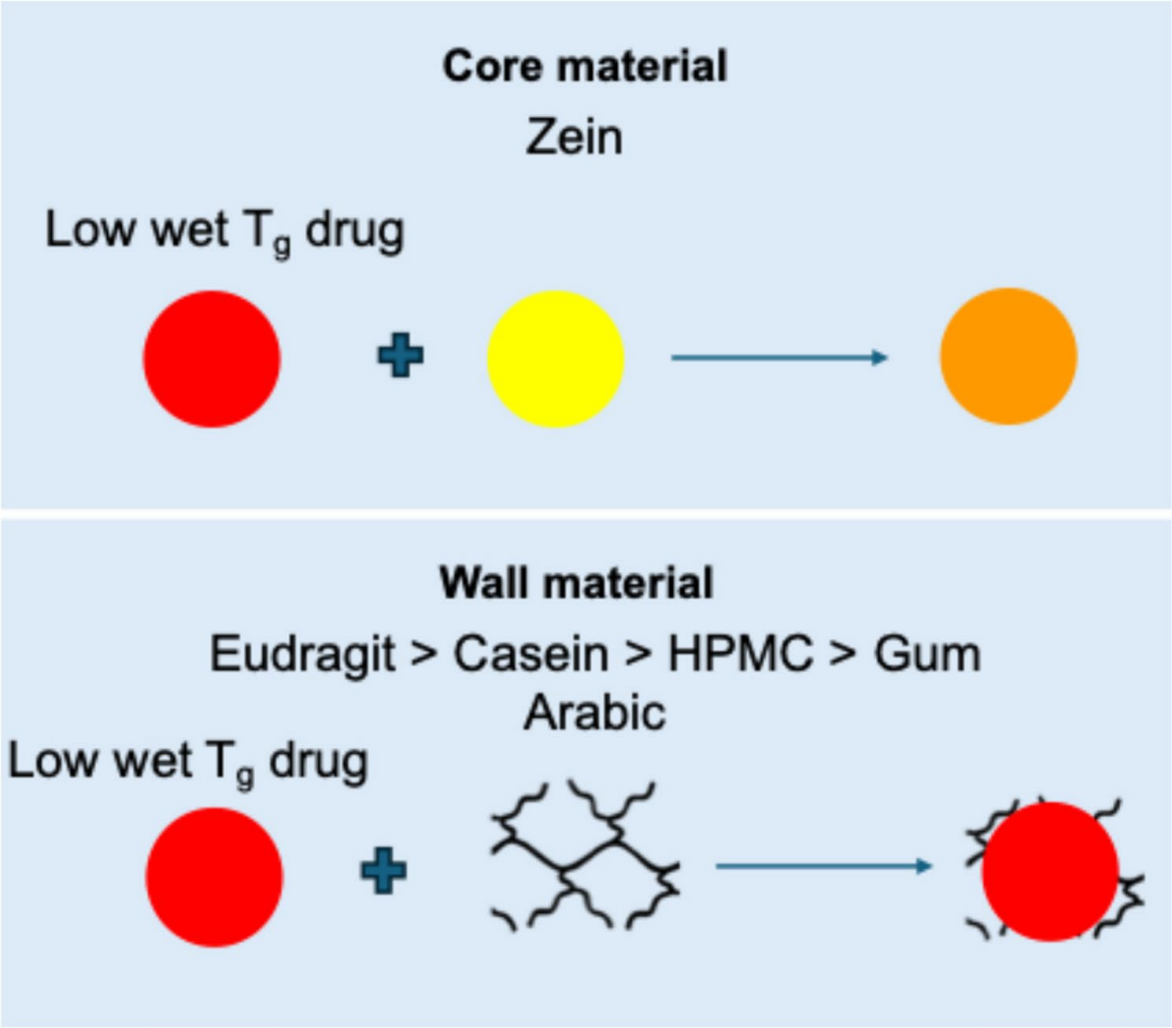
Schematic showing two classes of stabilizers

In our previous publication, coalescence and crystallization were identified as major particle growth pathways during thermal drying of amorphous drug nanoparticles (Narula et al. 2024). To prevent particle growth, stabilizers/excipients were chosen for the present study, which may be categorized as: core materials and wall materials (Fig. 7). This classification is supported by the stabilization efficiency and required mass ratios observed in our study (Fig. 2 and Tables S1 to S4). Core materials refer to excipients that are poorly soluble in the continuous phase and are miscible within the drug-rich phase such that they reside largely within the amorphous particle core, thereby effectively modifying particle properties such as T_g_ and rigidity. Core materials achieve complete stabilization at highly efficient, low concentrations (e.g., a 1:1 mass ratio). In contrast, wall materials remain primarily at the particle-liquid interface, acting as physical barriers that reduce mobility and contact via steric or electrostatic repulsion (Garti and Reichman 1993; Ueda and Taylor 2020). Because their stabilization mechanism depends on adequate surface coverage and barrier formation, wall materials inherently require substantially larger mass ratios (e.g., 1:10 to 1:20) to sufficiently coat and separate the soft particle surface and prevent coalescence during water removal, unless augmented by strong ionic interactions.

Core stabilizers, largely insoluble in the continuous phase, are different from solution partitioning stabilizers (Narula et al. 2024, 2026), which could exhibit substantial solubility in solution. Water-soluble excipients that were categorized as partitioning stabilizers in solution, such as HPMC, would act as wall materials during drying and require much higher quantities to effectively stabilize the particles upon removal of the continuous phase.

In our study, zein acted as a core material, which can partition into drug particles and increase their T_g_, and is therefore crucial for the solidification of nanoparticles and enhancing their thermal resistance (Feng et al. 2019). For example, it has been previously demonstrated that zein effectively co-assembles with FDP to form amorphous complexes driven by strong hydrophobic interactions (Ren et al. 2018), a stabilization mechanism that extends to other highly lipophilic drugs like CTZ and TNT.

Zein was specifically chosen for its high T_g_ of 164 °C (Di Gioia et al. 1999) and its miscibility with most poorly soluble drugs, effectively solidifying amorphous drug nanoparticles by imparting greater rigidity. As shown in Fig. 2, for all drugs investigated, zein effectively stabilized the nanoparticles at a low drug-to-polymer ratio of 1:1. However, from a drug release perspective, the use of water-soluble polymers as core materials is essential. pH-responsive polymers such as HPMCAS may potentially serve as effective core materials, enabling particle formation at low pH while ensuring fast dissolution at the site of drug absorption.

For wall materials, several key properties are required to effectively stabilize soft particles: they should dry quickly, form strong mechanical barriers, and have excellent emulsifying and coating abilities to ensure the stability of nanoparticles during both processing and storage (Labuschagne 2018). In this study, polymer and protein wall materials such as HPMC, gum Arabic, EUD, and casein were used. However, their ability in stabilizing amorphous drug nanoparticles differed greatly (Table S1-S4, Supporting Information): EUD and casein were able to stabilize particles at a low drug-to-polymer ratio of 1:1, whereas a much higher concentration was required for HPMC (1:20) and gum Arabic (1:10). EUD appeared to be most effective in stabilizing weakly basic drugs such as CTZ, BFZ, MCZ, and LTD, where drug-polymer ionic interactions potentially occurred. Nanoparticles stabilized through such potential ionic interactions exhibited better resistance to growth compared to drugs exhibiting relatively weak or no ionic interactions, such as ATZ and LPV (Fig. 3A). These results are consistent with our findings in the solution state, where ionic interactions, confirmed by ssNMR, also effectively stabilized amorphous drug nanoparticle in solution (Narula et al. 2024, 2026). For casein, all dried particles exhibited similar size (~ 400 nm), independent of the initial size of particles formed in solution. In addition, only a low amount of casein was required to effectively stabilize the particles, and its stabilizing effect was relatively independent of concentration. Therefore, for applications in which small particle sizes (< 300 nm) are not required, casein may present a suitable stabilizer choice.

During thermal drying of amorphous nanoparticle suspensions, the wall material must effectively prevent particle coalescence upon water removal. Our results suggest that stabilizer usage can be significantly reduced by leveraging ionic interactions between wall materials and drug particles, such as that observed for EUD stabilized weakly basic drug nanoparticles. In the absence of such interactions, good drying stability can be achieved either by increasing stabilizer concentration (e.g. gum Arabic, HPMC), or by employing a different stabilization mechanism (casein).

To produce nanoparticles, in addition to maintain drying stability (e.g. casein), a good solution stabilizer is needed to form small particle to start with (e.g. HPMCAS) (Narula et al. 2026). In this case, a combination of solution and drying stabilizers could be highly effective. For example, the Prud’homme group successfully produced re-dispersible lumefantrine nanoparticles, using HPMCAS to form nanoparticles with the active during flash nanoprecipitation and HPMC as a wall material to maintain drying stability (Armstrong et al. 2023).

### Enhanced membrane flux provided by amorphous drug nanoparticles

Our data suggested that the hydration state of amorphous drug nanoparticles, regardless of drug-excipient ionic interactions, did not impact particle re-dissolution rate and subsequent membrane permeability (Figs. 5 and 6). This is different from the observations reported by Bapat et al., where hydrophobic ion pairing between HPMCAS and the ionized drug led to slow drug release from ASDs (Bapat et al. 2024). Such difference could be attributed to the different polymer (HPMCAS and EUD) evaluated. Even if EUD would show the same behavior as HPMCAS, in this case, the small size of drug nanoparticles and longer time scale allowed for particle dissolution in these permeability experiments also allowed sufficient particle hydration. For many extremely poorly soluble drugs, increasing drug loading in ASDs without compromising its release is a challenging task. Our results suggested that dried high drug-loading amorphous drug nanoparticles can be used as an effective strategy to promote drug loading and achieve similar bioavailability as fast-releasing ASDs. Indeed, the effectiveness of dried amorphous drug nanoparticles was also reported for compound X (Purohit et al. 2024; Yu et al. 2025) and lumefantrine (Armstrong et al. 2023) with high in vivo bioavailability.

### Possible stability issues caused by drug crystallization

In this study, a batch thermal drying method (rotary evaporation) was employed; therefore, drying time was dependent on batch size. For example, the average drying time increased from approximately 13 min for a 2 mL sample to more than 30 min for a 6 mL sample. This extended thermal exposure adversely affected nanoparticle stability and, in the case of CTZ-EUD nanoparticles, promoted drug crystallization during drying. These observations indicate that, when batch thermal drying methods are used, formulation development should account not only for composition but also for scale-dependent process parameters, particularly drying temperature and drying time. Alternatively, rapid drying methods such as spray drying could be used to reduce thermal exposure time.

In addition, the stabilizer of choice must sufficiently interact with the drug within the particle phase, as the formation of a neat drug core is highly undesirable. Insufficient drug-polymer interactions substantially increase the risk of drug crystallization. In formulation development, if a surface stabilizer that does not partition into the particles is used, it should be combined with a partitioning stabilizer to minimize crystallization risk.

In the context of scaled applications and manufacturing, long-term storage stability of the dried intermediate is a critical consideration that might also affect drug crystallization in nanoparticles. Given the drug-rich nature of some amorphous nanoparticles, low polymer concentrations in the particle phase may be insufficient to effectively inhibit drug crystallization over extended storage periods. For rapid crystallizing drugs, this issue can be mitigated by incorporating a core material polymer, such as HPMCAS, into the particle phase. This strategy has previously been used to successfully produce stable amorphous nanoparticles of lumefantrine and delamanid (Armstrong et al. 2023; Caggiano et al. 2023). While the present study establishes the feasibility of producing and stabilizing dried amorphous drug nanoparticles, the translation of this intermediate into a final solid dosage form (e.g., tablets or capsules) requires further investigation (Lee 2003). The mechanical stresses of downstream processing, particularly compression, may introduce additional crystallization risks, potentially requiring high-T_g_ core stabilizers to maintain particle rigidity (Tortorella et al. 2021; Armstrong et al. 2023). From a bioperformance perspective, provided that the final dosage form is optimized for rapid disintegration and re-dispersion, the formulated nanoparticles are expected to retain their permeability advantages. By acting as highly concentrated drug reservoirs within the gastrointestinal tract, they can maintain maximum supersaturation (Indulkar et al. 2016) and enhance absorption via the particle drifting effect (Sugano 2010; Yang et al. 2024).

## Conclusion

Thermal drying stability is essential to the production of robust amorphous drug loaded nanoparticles. Drying stability is impacted by several factors such as choice of excipients used as stabilizer, batch scale, drug-excipient interactions and optimization of drying process parameters. Our detailed study focusing on stabilizers showed that core materials provided effective stabilization at low concentrations, whereas wall materials required either strong ionic interactions with the drug, increased stabilizer levels, or alternative stabilization mechanisms such as forming Pickering emulsion, to achieve successful stability during thermal drying. Formulation stability in batch drying processes was found to be strongly influenced by batch size, primarily due to crystallization-related challenges. This may be mitigated through process modification or formulation optimization. The dried nanoparticles retained their permeability advantages regardless of ionic interactions or drying methods. Collectively, these findings confirm amorphous drug nanoparticles as a promising alternative to conventional amorphous solid dispersion formulations, enabling higher drug loading while maintaining comparable bioavailability.

## Supplementary Information

Supplementary Material 1.

## Data Availability

All data generated and analyzed during this project are included in this article and its supplementary information file. Raw data are also available upon request.

## Abbreviations

ASD: Amorphous solid dispersion
ATZ: Atazanavir
BFZ: Bifonazole
CTZ: Clotrimazole
DLS: Dynamic light scattering
DMSO: Dimethyl sulfoxide
DSC: Differential scanning calorimetry
EDX: Energy-dispersive X-ray spectroscopy
EUD: Eudragit^®^ L100
FDP: Felodipine
FFT: Fast Fourier transform
HAADF: High angle annular dark-field
HPLC: High-performance liquid chromatography
HPMC: Hydroxypropyl methylcellulose
KTZ: Ketoconazole
LLPS: Liquid-liquid phase separation
LTD: Loratadine
LPV: Lopinavir
MCZ: Miconazole
PLM: Polarized light microscopy
PVDF: Polyvinylidene fluoride
ssNMR: Solid-state nuclear magnetic resonance
STEM: Scanning transmission electron microscopy
T_g_: Glass transition temperature
TNT: Tolnaftate
TEM: Transmission electron microscopy
