# Supplementary material for: Drying stability and permeability advantages of amorphous drug nanoparticles
Source: AAPS Open. 2026 Jul 24;12(1):46. doi: 10.1186/s41120-026-00185-z (PMC13400673; doi:10.1186/s41120-026-00185-z)
Supplement: Supplementary file 1 — Supplementary Material 1. [file 41120_2026_185_MOESM1_ESM.docx]

Supporting Information for the manuscript entitled

Drying stability and permeability advantages of amorphous drug nanoparticles

Akshay Narula^1†^, Ajay Lale^1†^, Paroma Chakravarty^2^, Na Li^1, 3, 4*^

^1^Department of Pharmaceutical Sciences, University of Connecticut, 69 North Eagleville Road Unit 3092, Storrs, CT 06269, United States

^2^Synthetic Molecule Pharmaceutical Sciences, Genentech, Inc., San Francisco, CA 94080, United States

^3^Institute of Materials Science, University of Connecticut, 97 North Eagleville Road Unit 3136, Storrs, CT 06269, United States

^4^Department Chemical & Biomolecular Engineering, University of Connecticut, 191 Auditorium Road, Unit 3222, Storrs, CT 06269, United States

^†^A.N. and A.L. contributed equally to this work

*Corresponding Author Email: [lina@uconn.edu](mailto:lina@uconn.edu)


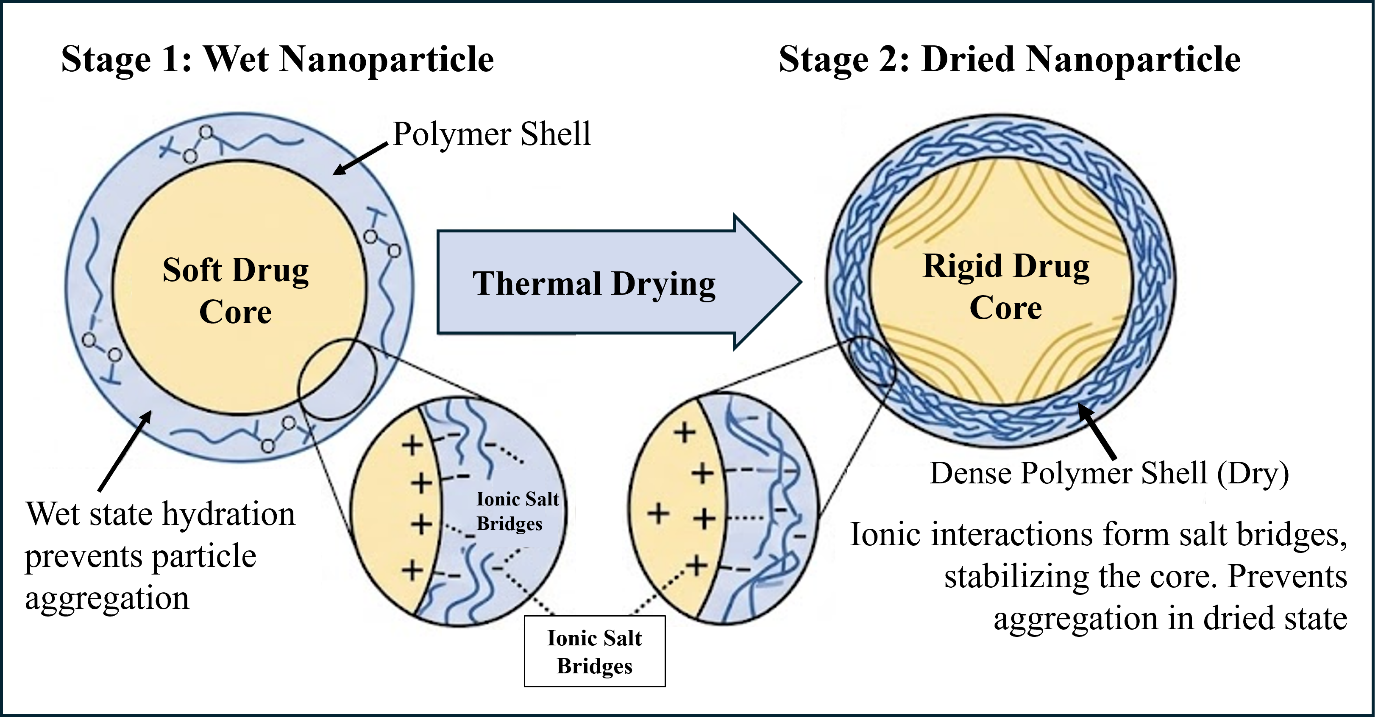


Figure S1. Mechanism of nanoparticle stabilization during thermal drying. Intermolecular salt bridges form between the protonated weakly basic drug (+) and the deprotonated anionic polymer (-), creating a rigid polymer shell that prevents particle coalescence in the dried state.


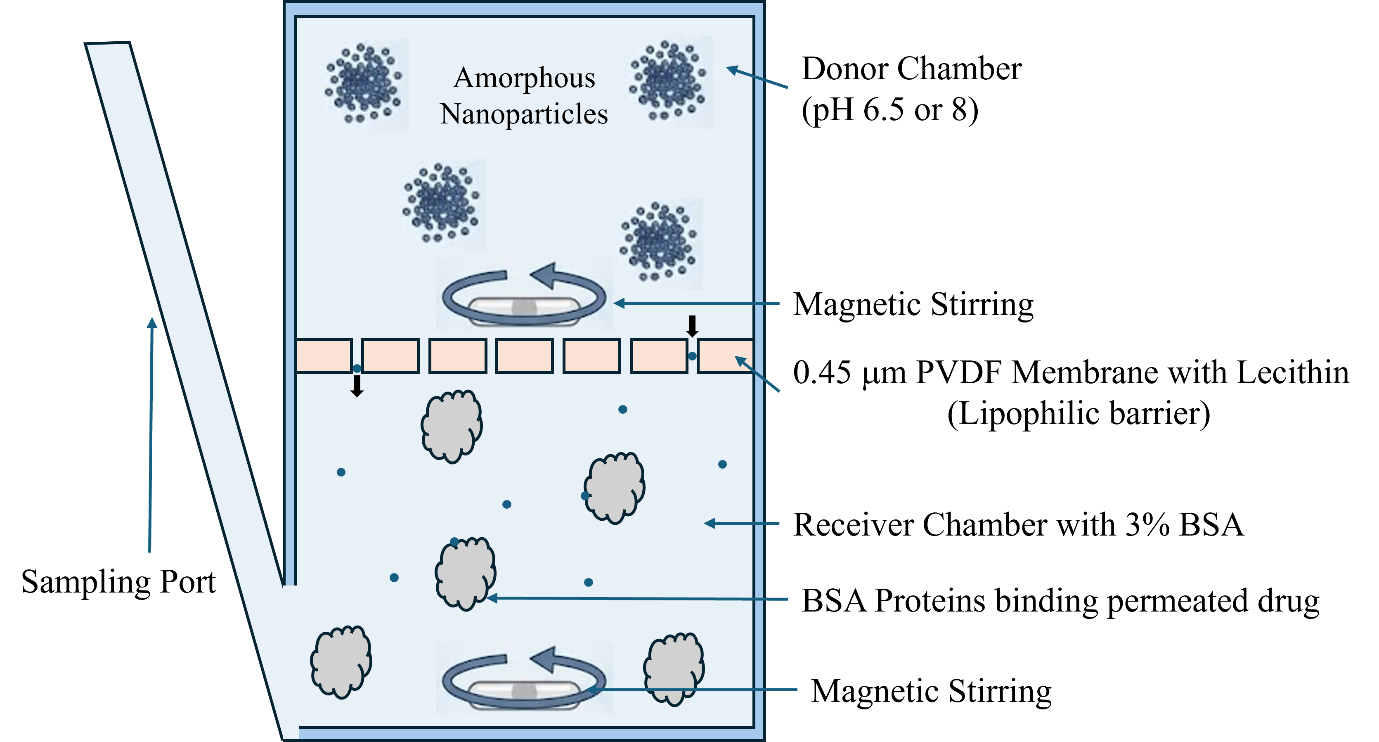


Figure S2. Franz Diffusion Cell Setup


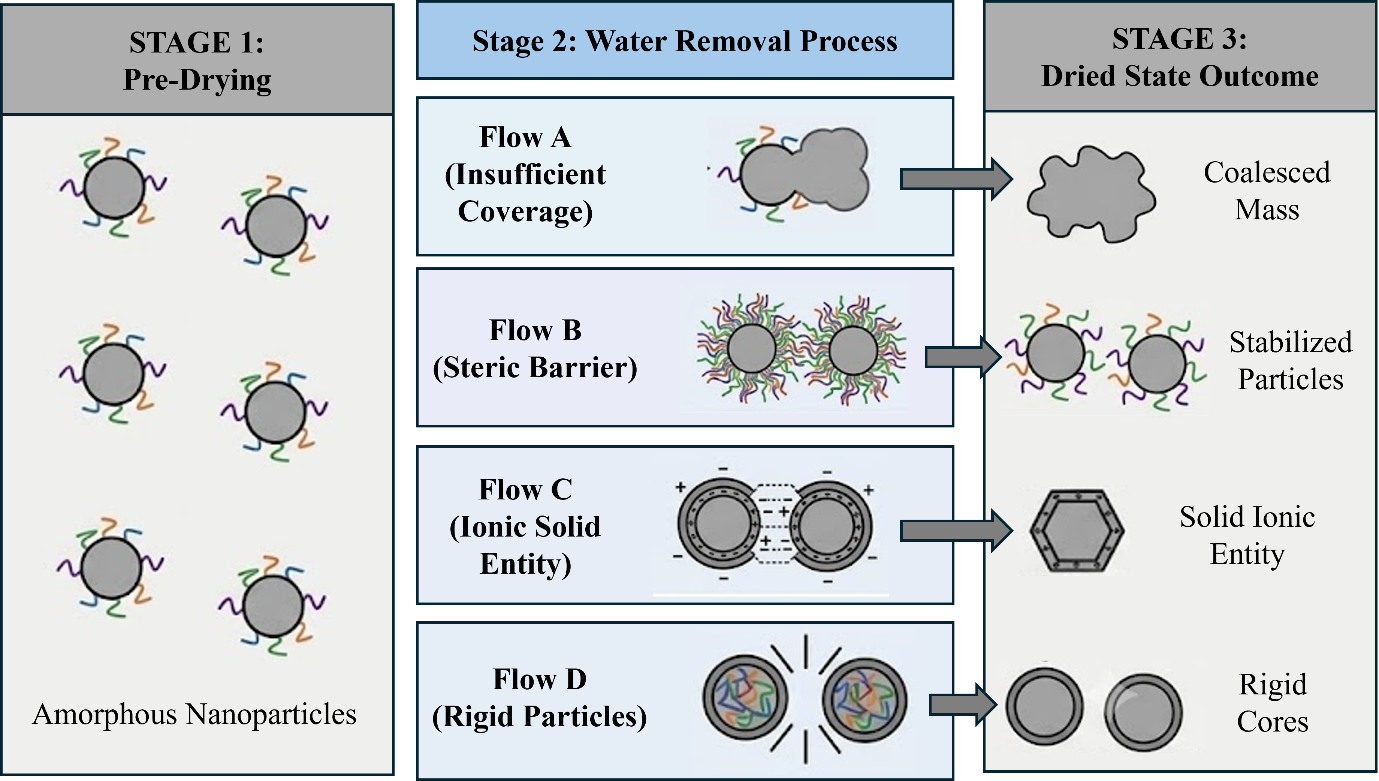


Figure S3. Schematic illustrating the impact of stabilizer selection on nanoparticle behavior post-drying

Table S1. Particle size at drug: Eudragit ratios pre- and post-drying

| System | Drug: Stabilizer | Before drying | After drying |
| --- | --- | --- | --- |
| CTZ: EUD | 1:1 | 247±9.05 | 269.2± 22.34 |
| FDP: EUD | 1:1 | 288.53±53.16 | 586.03±15.68 |
|  | 1:2 | 271.13±11.93 | 686.7±98.22 |
|  | 1:5 | 239.93±3.65 | 618.26±128.79 |
|  | 1:10 | 255.53±5.11 | 696.76±12.61 |
|  | 1:20 | 338.96±8.28 | 637.03±31.08 |
| TNT: EUD | 1:1 | 409.9±104.46 | 547.26±52.49 |
|  | 1:2 | 374.26±26.25 | 619.4±46.84 |
|  | 1:5 | 393.2±1.70 | 564.46±89.2 |
|  | 1:10 | 357.16±12.85 | 597.3±59.14 |
|  | 1:20 | 407.96±20.57 | 629.06±29.84 |

Table S2. Particle size at drug: HPMC ratios pre- and post-drying

| System | Drug: Stabilizer | Before drying | After drying |
| --- | --- | --- | --- |
| CTZ: HPMC | 1:1 | 321 | 4504* |
|  | 1:2 | 291.6 | 1137* |
|  | 1:5 | 321.7 | 1308* |
|  | 1:10 | 272.3±22.96 | 1852.33±898.23* |
|  | 1:20 | 374.1±14.36 | 351.4±12.73 |
| FDP: HPMC | 1:1 | 279.6 | 47420* |
|  | 1:2 | 293.6 | 2682* |
|  | 1:5 | 262.5 | 1140* |
|  | 1:10 | 308.4±97.51 | 488.76±141.41* |
|  | 1:20 | 291.33±10.65 | 285.33±43.29 |
| TNT: HPMC | 1:1 | 372.63±66.17 | 626.16±63.81 |
|  | 1:2 | 309.13±12.75 | 611.53±70.78 |
|  | 1:5 | 366.2 | 696.1* |
|  | 1:10 | 300.03±22.10 | 481.73±78.45 |
|  | 1:20 | 378.46±41.39 | 502±52.19 |

*poor quality data

Table S3. Particle size at drug: Gum Arabic ratios pre- and post-drying

| System | Drug: Stabilizer | Before drying | After drying |
| --- | --- | --- | --- |
| CTZ: Gum Arabic | 1:1 | 307.4 | 1070* |
|  | 1:2 | 312.9 | 571.2 |
|  | 1:5 | 774.6 | 1361* |
|  | 1:10 | 632.83±19.82 | 777.66±77.84 |
|  | 1:20 | 442.76±4.10 | 833.16±70.14 |
| FDP: Gum Arabic | 1:1 | 406.1 | 1028* |
|  | 1:2 | 362.2 | 976.5 |
|  | 1:5 | 554.4 | 820 |
|  | 1:10 | 615.96±81.24 | 817.03±11.24 |
|  | 1:20 | 405.4±9.85 | 724±77.53 |
| TNT: Gum Arabic | 1:1 | 334 | 2183* |
|  | 1:2 | 292.9 | 1946* |
|  | 1:5 | 618.8 | 1109* |
|  | 1:10 | 580.03±30.12 | 835.3±36.81 |
|  | 1:20 | 505.66±96.62 | 630.83±87.32 |

*poor quality data

Table S4. Particle size at drug: Casein ratios pre- and post-drying

| System | Drug: Stabilizer | Before drying | After drying |
| --- | --- | --- | --- |
| CTZ: Casein | 1:1 | 236.53±22.21 | 414.23±33.92 |
|  | 1:2 | 275.36±57.04 | 444.13±69.72 |
|  | 1:5 | 228.2±33.08 | 572.23±165.57* |
|  | 1:10 | 185.46±6.86 | 341.93±75.31* |
|  | 1:20 | 143.56±4.35 | 116.8±11.91* |
| FDP: Casein | 1:1 | 232.9±33.56 | 404.13±27.47 |
|  | 1:2 | 267.4±16.99 | 474.76±38.44 |
|  | 1:5 | 281±33.47 | 385.33±33.47 |
|  | 1:10 | 291.66±59.37 | 306.66±16.49 |
|  | 1:20 | 284.06±10.4 | 251.36±3.85 |
| TNT: Casein | 1:1 | 337.86±27.79 | 383.2±6.5 |
|  | 1:2 | 328.86±89.53 | 442.23±44.21 |
|  | 1:5 | 301.13±34.12 | 425.03±42.91 |
|  | 1:10 | 266.76±42.66 | 348.63±30.39 |
|  | 1:20 | 264.6±10.21 | 358.46±15.69 |

*poor quality data
